# Green Synthesis of Silver Nanoparticles Using Zingiber officinale and Ocimum gratissimum Formulation for Its Anti-inflammatory and Antidiabetic Activity: An In Vitro Study

**DOI:** 10.7759/cureus.58098

**Published:** 2024-04-12

**Authors:** Aakashwar Anandachockalingam, Rajeshkumar Shanmugam, Iadalin Ryntathiang, Mukesh Kumar Dharmalingam Jothinathan

**Affiliations:** 1 Nanobiomedicine Laboratory, Centre for Global Health Research, Saveetha Medical College and Hospital, Saveetha Institute of Medical and Technical Sciences, Saveetha University, Chennai, IND; 2 Centre for Global Health Research, Saveetha Medical College and Hospital, Saveetha Institute of Medical and Technical Sciences, Saveetha University, Chennai, IND

**Keywords:** antidiabetic, anti-inflammatory, silver nanoparticles (agnps), eco-friendly, green synthesis

## Abstract

Aim

This study aims to synthesize silver nanoparticles (AgNPs) using herbal formulations derived from *Zingiber officinale* (ginger) and *Ocimum gratissimum* and to evaluate their anti-inflammatory and antidiabetic activities in vitro.

Methods

The synthesis of AgNPs was performed using *Z. officinale *and *O. gratissimum*, and the AgNPs were confirmed by analyzing their ultraviolet-vis spectra. The anti-inflammatory activity was assessed using two assays, specifically the bovine serum albumin (BSA) denaturation assay and the egg albumin (EA) denaturation assay. The antidiabetic activity was assessed using the α-amylase inhibitory assay and the β-glucosidase inhibitory assay.

Results

This study evaluated the anti-inflammatory and antidiabetic activities of green-synthesized AgNPs using *Z. officinale* and *O. gratissimum*. The maximum absorption peak was observed for AgNPs at ~433 nm (wavelength). In the BSA denaturation assay, 78% inhibition was observed at a concentration of 50 μl. Similarly, in the EA denaturation assay, an inhibition of 74% was observed at the same concentration compared to the standard. In terms of antidiabetic activity, when compared to the standard at a concentration of 50 μl, the α-amylase inhibitory assay and the β-glucosidase inhibitory assay showed approximately 78% and 80% inhibition, respectively.

Conclusion

The use of *Z. officinale* and *O. gratissimum* extracts for the synthesis of AgNPs using a green synthesis method presents a sustainable and environmentally friendly approach. The synthesized AgNPs demonstrated significant anti-inflammatory and antidiabetic efficacy, suggesting their potential application in pharmaceuticals for treating diabetes and inflammation. Further research is necessary to investigate the effectiveness and safety of these substances in humans and to understand their underlying mechanisms of action.

## Introduction

Nanotechnology is an advancing technology applied in several scientific domains such as biology, chemistry, and material science [1]. Nanoparticles (NPs), produced via several techniques, have attracted attention because of their unique attributes [2]. In addition, there has been an increased focus on metal-based NPs, such as zinc sulfide (ZnS), selenium (Se), gold (Au), zinc oxide (ZnO), and silver (Ag), because of their unique features and specific qualities [3,4]. Out of these several types of NPs, silver NPs (AgNPs) have shown promise in biomedical applications because of their antimicrobial, antioxidant, anti-inflammatory, antidiabetic, antiviral, cytotoxic, and anticancer activities. Furthermore, AgNPs are easiest to produce and handle due to their simplicity [5-7]. AgNPs generated through green synthesis have proven to be highly notable for their environmental friendliness and economic viability. Green synthesis methods, involving the use of microbes or medicinal plants, particularly those with therapeutic characteristics, have gained prominence for producing AgNPs due to their environmental friendliness and economic viability [8]. Phytochemicals such as alkaloids, terpenes, saponins, phenols, alcohols, and proteins are found in plant extracts. These compounds can act as both capping agents and reducing agents. This facilitates the enlargement and structural improvement of nanomaterials, making them more applicable in pharmacological and biological research endeavors [9].

Inflammation is an innate physiological reaction to harmful factors such as infections, injuries, or irritants. It is a vital mechanism that safeguards and promotes the healing of our bodies. Nevertheless, an abundance or extended duration of inflammation can result in discomfort and various health complications. Therefore, it is vital to acquire a thorough comprehension of the various anti-inflammatory medications currently available on the market due to the rising occurrence of pain-related illnesses and the intricate nature of healthcare interventions. Nonsteroidal anti-inflammatory medicines have been associated with heartburn, allergies, dizziness, headaches, and stomach ulcers [10,11]. Simultaneously, diabetes mellitus is a systemic metabolic condition that impacts multiple organs in the body and affects individuals in both developed and developing nations. This disease affects approximately 25% of the global population. Diabetic patients experience impaired glucose utilization in the body because of inadequate insulin release from the β-cells of the pancreas. Diabetes causes significant damage to vital organs such as the pancreas, kidneys, and liver. Increased glucose catabolism causes a decrease in hepatic glycogen levels, which eventually leads to liver damage. Food behaviors and hereditary factors mostly influence diabetes [12,13]. Hence, there is a growing call to explore herbal therapies due to their lesser adverse effects compared to allopathic treatments. *Zingiber officinale* belongs to the Zingiberaceae family and is commonly known as ginger, which has been recognized since ancient times for its medicinal properties. Ginger has a diverse array of pharmacological effects, including gastroprotective, antidiabetic, antioxidant, anti-inflammatory, and antibacterial properties. In addition, ginger possesses shogaols, zingiberene, gingerols, and paradols, which contribute to the antioxidant properties of *Z. officinale* [14,15]. Similarly, *Ocimum gratissimum* is a herbaceous plant that belongs to the Labiatae family. The plant species is native to tropical regions, particularly West Africa and India, and can be found in Nigeria’s savannah and coastal regions [16]. Furthermore, it is highly regarded as a medicinal plant because of its notable therapeutic properties. It has been applied in different areas of medicine, specifically oral medicine, because of its anti-inflammatory, antibacterial, anti-ulcer, and analgesic features [17].

This study aims to evaluate the anti-inflammatory and antidiabetic activity of AgNPs using a green synthesis method involving *Z. officinale* and *O. gratissimum* herbal formulations. The bovine serum albumin (BSA) denaturation assay and the egg albumin (EA) denaturation assay are the two assays used to evaluate anti-inflammatory efficacy. Furthermore, the antidiabetic activity was evaluated using two distinct techniques: the α-amylase inhibition assay and the β-glucosidase inhibition assay.

## Materials and methods

This study was conducted at Saveetha Dental College and Hospitals, a division of Saveetha Institute of Medical and Technical Sciences (SIMATS) and Saveetha University, Chennai, India.

Preparation of the herbal formulation

One gram of* Z. officinale* (ginger) and one gram of *O. gratissimum* were accurately weighed and combined with 100 mL of distilled water. Subsequently, the prepared mixture was heated to 60°C for 20 minutes using a heating mantle while continuously stirring. The boiled extract was then cooled and filtered through muslin fabric, and the filtrate was collected. The filtrate was further purified by filtering through Whatman No. 1 filter paper to eliminate any remaining particles. The filtrate obtained, which contained the extract, was subsequently used for the synthesis of NPs.

Instruments

The instruments used in this study included a Labman Scientific ultraviolet (UV)-visible spectrophotometer (200-600 nm, Chennai, India), a hot air oven (Techno/EBI, Techno Instruments Company, Bengaluru, India), a global digital pH meter (Techno/EBI, Techno Instruments Company), a water bath (Techno/EBI, Techno Instruments Company), centrifugation (REMI C-24 Plus, Chennai, India), and a REMI 1MLH stirrer (REMI Lab World, Mumbai, India).

Green synthesis of AgNPs

**Figure 1:**
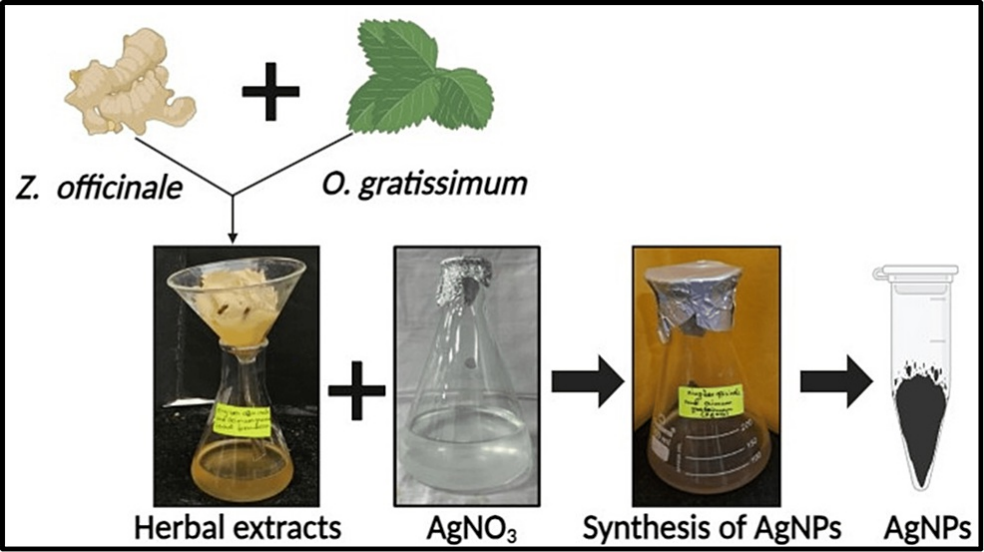
Diagrammatic representation of the green synthesis of AgNPs AgNO_3_, silver nitrate; AgNPs, silver nanoparticles

The green synthesis method was used for the synthesis of AgNPs mediated by *Z. officinale* and* O. gratissimum* extracts. A 1 mM of silver nitrate (AgNO_3_) was mixed with 80 mL of distilled water. Following this, 20 mL of a filtered herbal formulation extract was introduced into the solution. The mixtures were then placed in a magnetic stirrer at a speed of 800 rpm for 24-48 hours. The color change from light brown to dark brown indicates the successful synthesis of AgNPs. Figure 1 displays a diagrammatic representation of the green synthesis of AgNPs.

Characterization of AgNPs 

Using a Labman Scientific UV-visible spectrophotometer, the UV-vis spectra of the AgNPs were observed after 24 hours within the wavelength range of 200-600 nm. Afterward, the NPs were centrifuged at a speed of 8,000 rpm for 10 minutes. Centrifugation was used to separate the AgNP pellet from the supernatant. The supernatant was discarded, while the pellet was placed in a sealed Eppendorf tube (Eppendorf SE, Hamburg, Germany) and stored for further experiments.

Evaluation of anti-inflammatory effect

The efficacy of the anti-inflammatory agent was assessed using two assays, specifically the BSA denaturation assay and the EA denaturation assay, as reported in a previous study by Harris et al. [18].

BSA Denaturation Assay

A solution of BSA with a volume of 0.45 mL was mixed with 0.05 mL of *Z. officinale* (ginger) and *O. gratissimum *extracts at concentrations ranging from 10 to 50 µg/mL. The pH was adjusted to a precise value of 6.3. The sample was then kept at room temperature for 20 minutes and then transferred to a water bath at 55°C for 30 minutes. Dimethyl sulfoxide was used as the control group, whereas diclofenac sodium was employed as the standard group. Subsequently, at 660 nm wavelength, the samples were evaluated using a spectrophotometer. The percentage of inhibition of protein denaturation was calculated using the following formula:

$Percentage \hspace{00.2cm} of \hspace{00.2cm} inhibition= \left ( Absorbance \hspace{00.2cm} of \hspace{00.2cm}control \right )- \left ( Absorbance \hspace{00.2cm} of \hspace{00.2cm}sample \right ) \setminus \left (Absorbance \hspace{00.2cm} of \hspace{00.2cm}control \right )\times 100$

EA Denaturation Assay

To perform the EA denaturation test, 2.8 mL of phosphate buffer was mixed with 0.2 mL of freshly acquired EA. Various concentrations (ranging from 10 to 50 µg/mL) of* Z. officinale* (ginger) and* O. gratissimum* extract (5 mL) was added to the mixture. The pH was modified to 6.3, and the mixture was kept at room temperature for 20 minutes before incubation in a water bath at 55°C for 30 minutes. Diclofenac was employed as the standard group, whereas dimethyl sulfoxide was used as the control group. The absorbance was measured at 660 nm. The percentage of inhibition of protein denaturation was calculated using the following formula:

$(Percentage \hspace{00.2cm} of \hspace{00.2cm} inhibition= \left ( Absorbance \hspace{00.2cm} of \hspace{00.2cm}control \right )- \left ( Absorbance \hspace{00.2cm} of \hspace{00.2cm}sample \right ) \setminus \left (Absorbance \hspace{00.2cm} of \hspace{00.2cm}control \right )\times 100$

Anti-diabetic activity

α-Amylase Assay

The study on inhibiting α-amylase assay in vitro followed the Agarwal et al. method [19]. A 100 µL of α-amylase solution (1 U/mL) was combined with different amounts of NPs ranging from 10 to 50 µL and left to incubate at room temperature for 30 minutes. A starch solution containing 100 µL of 1% (w/v) was added. The sample was then kept at room temperature for 10 minutes. A 100 µL volume of a 96 mM solution of 3,5-dinitrosalicylic acid (DNSA reagent) was added to the mixture to stop the reaction and heated in a water bath for five minutes. Sodium phosphate buffer (pH of 6.9) was used as a control by replacing an equal quantity of enzyme extract. The reading was measured at a wavelength of 540 nm. The experiment was conducted in triplicate. For the positive control, acarbose was used. The percentage of inhibition of α-amylase was calculated using the following formula:

$Percentage \hspace{00.2cm} of \hspace{00.2cm} inhibition= \left ( Absorbance \hspace{00.2cm} of \hspace{00.2cm}control \right )- \left ( Absorbance \hspace{00.2cm} of \hspace{00.2cm}sample \right ) \setminus \left (Absorbance \hspace{00.2cm} of \hspace{00.2cm}control \right )\times 100$

β-Glucosidase Assay

To assess the β-glucosidase enzyme inhibition experiment, the procedure followed a protocol modified from a previous study [20]. Starch solution (2% w/v maltose or sucrose) was mixed with AgNPs in quantities ranging from 10 to 50 µg/mL. Subsequently, Tris buffer (0.2 M) was introduced into the sample at pH 8.0. The reaction was then incubated at 37°C for five minutes. Subsequently, 1 mL of β-glucosidase enzyme (1 U/mL) was added. Then, the reaction was allowed to proceed at room temperature for 40 minutes. To stop the reaction, 2 mL of 6 N HCl was added. Acarbose served as the positive control. The absorbance was measured at a wavelength of 540 nm. The percentage of inhibition of β-glucosidase was calculated using the following formula:

$Percentage \hspace{00.2cm} of \hspace{00.2cm} inhibition= \left ( Absorbance \hspace{00.2cm} of \hspace{00.2cm}control \right )- \left ( Absorbance \hspace{00.2cm} of \hspace{00.2cm}sample \right ) \setminus \left (Absorbance \hspace{00.2cm} of \hspace{00.2cm}control \right )\times 100$

## Results

UV-visible spectroscopy

**Figure 2:**
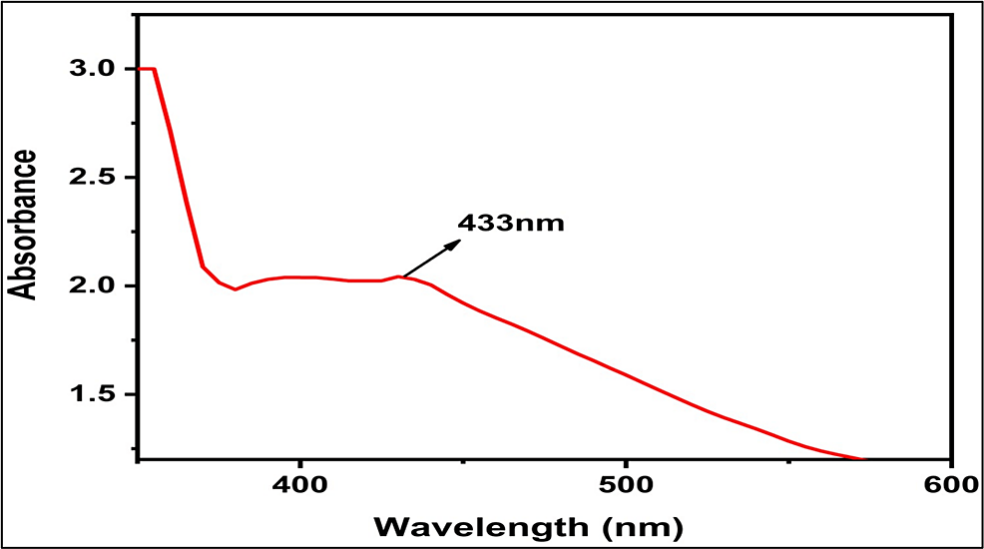
UV-visible spectrum of green-synthesized AgNPs AgNPs, silver nanoparticles; UV, ultraviolet

This study confirmed the AgNP reaction by examining their UV-vis spectra, as shown in Figure 2. After 24 hours post-synthesis, the maximum absorption peak at ~433 nm was observed, suggesting the occurrence of contact between the metal and the formulation extracts, leading to the synthesis of AgNPs.

Anti-inflammatory activity

BSA Denaturation Assay

**Figure 3:**
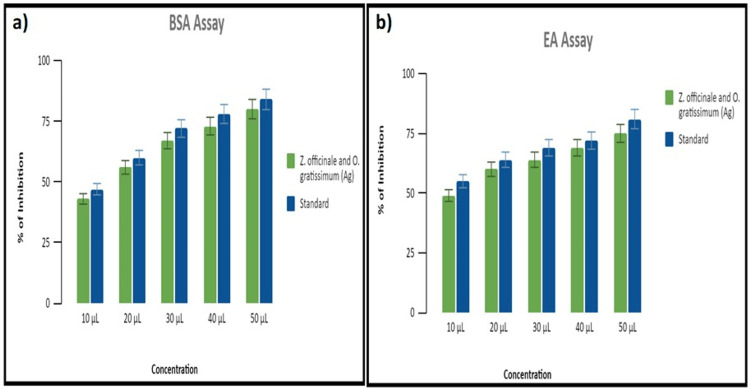
Anti-inflammatory activity of green-mediated AgNPs using (a) BSA denaturation assay and (b) EA denaturation assay AgNPs, silver nanoparticles; BSA, bovine serum albumin; EA, egg albumin

The AgNPs mediated by *Z. officinale* and *O. gratissimum* formulations demonstrated distinct levels of inhibition at various concentrations ranging from 10 to 50 µg/mL. The inhibition of *Z. officinale*- and *O. gratissimum*-mediated AgNPs were 45%, 56%, 65%, 70%, and 78%. In comparison, the percentages of diclofenac sodium were 49%, 60%, 70%, 75%, and 81%. Although the *Z. officinale*- and* O. gratissimum*-mediated AgNPs showed anti-inflammatory properties at all concentrations, the standard consistently displayed significantly greater suppression of BSA denaturation over the entire range of concentrations (Figure 3a).

EA Denaturation Assay

Figure 3b displays the outcomes of the EA denaturation assay, revealing that the AgNPs facilitated by the *Z. officinale* and *O. gratissimum* formulations exhibited varying levels of inhibition at concentrations ranging from 10 to 50 µg/mL. The percentages of inhibition of synthesized AgNPs were 49%, 58%, 60%, 67%, and 74%. In comparison, diclofenac sodium exhibited inhibition percentages of 55%, 60%, 69%, 72%, and 81%. Although the formulation of AgNPs exhibited anti-inflammatory activities at all concentrations, the standard consistently demonstrated considerably stronger inhibition of EA denaturation among all concentrations.

Anti-diabetic activity

α-Amylase Assay

**Figure 4:**
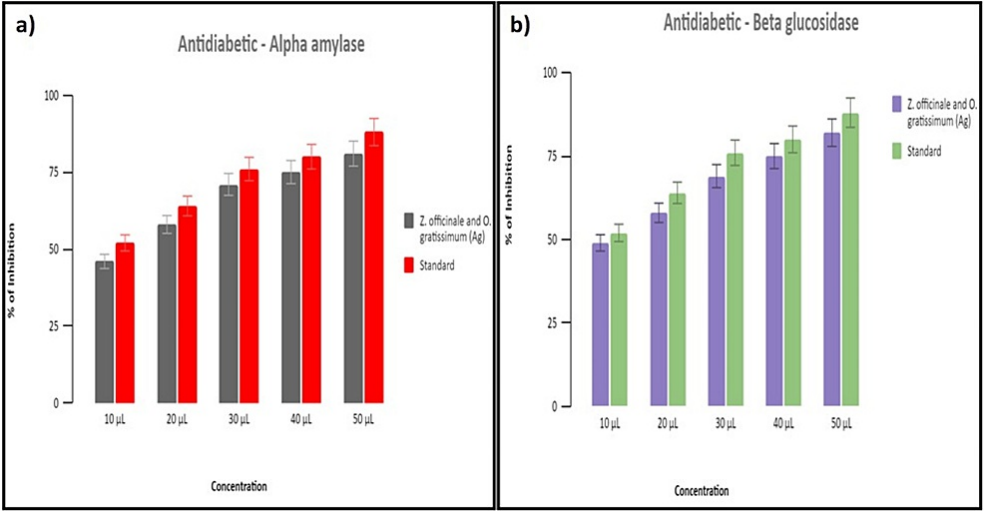
Antidiabetic activity of AgNPs using (a) α-amylase inhibitory assay and (b) β-glucosidase inhibitory assay AgNPs, silver nanoparticles

The AgNPs produced by *Z. officinale *and *O. gratissimum* showed inhibition that increased with concentrations ranging from 10 to 50 µg/mL. In Figure 4a, the inhibition percentages are shown as 48%, 60%, 68%, 75%, and 78%. In comparison, acarbose demonstrated inhibition ranging between 51% and 84% at the same concentrations. These findings suggest that AgNPs can control the activity of α-amylase, and the herbal formulation affects their ability to suppress this activity.

β-Glucosidase Assay

The AgNPs produced by *Z. officinale* and *O. gratissimum* showed an increase in inhibition that was dependent on the concentration. The inhibition percentages were 49%, 62%, 70%, 74%, and 80%, respectively, at varying concentrations (ranging from 10 to 50 µg/mL). In contrast, acarbose exhibited varying percentages, ranging from 51% to 81% when administered at equivalent doses. These findings suggest that AgNPs can regulate β-glucosidase activity, while the herbal formulation affects its ability to suppress this activity (Figure 4b).

## Discussion

This study aimed to investigate the anti-inflammatory properties and antidiabetic efficacy of AgNPs synthesized using a green synthesis method involving *Z. officinale* and *O. gratissimum* herbal formulations. This study involved the synthesis of AgNPs and their characterization using UV-visible spectrophotometry and visual observation. The anti-inflammatory activity was assessed using the BSA denaturation assay and the EA denaturation assay. Similarly, for antidiabetic activity, α-amylase and β-glucosidase enzymes were assessed. This research seeks to make substantial advances in the field of green nanotechnology and its applications in therapeutic science.

According to Ramteke et al., the UV absorption peak of AgNPs is commonly found between the wavelengths of ~300 and ~900 nm [21]. The *Z. officinale*- and *O. gratissimum*-mediated AgNPs show clear absorption peaks in the UV range, specifically at ~433 nm. This observation affirms the presence of AgNP synthesis in plant extracts.

Furthermore, it is crucial to understand that AgNPs have undergone a thorough examination regarding their antibacterial characteristics. However, new investigations have also revealed their ability to reduce inflammation. Lan Chi et al. reported the anti-inflammatory properties of AgNPs produced using an aqueous kernel extract of *Azadirachta indica* [22]. The study discovered that these AgNPs demonstrated anti-inflammatory properties similar to those of conventional medications administered at a dose of 100 μg mL^−1^. This indicates the potential effectiveness of AgNPs in controlling inflammation. Shehensha and Jyothi found that AgNPs, when exposed to reactive oxygen species, exhibit both anti-inflammatory and pro-inflammatory effects. The different results highlight the need for a comprehensive understanding of the mechanisms and conditions under which AgNPs are used in anti-inflammatory applications [23]. Furthermore, Mohamed et al. performed a research study on the synthesis of AgNPs using cinnamon oil and found that they exhibited strong anti-inflammatory effects similar to those of diclofenac sodium, a commonly used anti-inflammatory medication [24]. These findings provide further evidence for the potential of AgNPs as highly effective anti-inflammatory medicines.

In addition, the antidiabetic effectiveness of AgNPs was assessed by conducting in vitro studies on α-amylase and β-glucosidase enzymes, which play an important role in diabetes treatment. α-amylase is an enzyme that facilitates the breakdown of complex carbohydrates through hydrolysis, resulting in the production of simpler sugars, such as glycogen. Suppression of α-amylase activity can slow down the rate at which carbohydrates are converted into glucose. After consuming a meal, there is a delayed release of glucose into the bloodstream. The inhibitory effect of green-synthesized AgNPs on α-amylase activity has been studied. When the NPs are exposed to α-amylase, they can interact with the enzyme and delay its normal action, leading to a reduction in the breakdown of carbohydrates and the subsequent release of glucose [25]. β-glucosidase is an enzyme that occurs during carbohydrate degradation. The enzyme’s role is to catalytically break down complex carbohydrates (such as disaccharides) into simpler sugars (such as glucose) that may be absorbed into the bloodstream. By suppressing the function of β-glucosidase, the conversion of complex carbohydrates into glucose can be decelerated, leading to a reduction in the rate of glucose absorption in the intestine. The inhibitory effect of AgNPs generated using green methods on the activity of the β-glucosidase enzyme has also been studied [26]. These NPs possess the capacity to attach to β-glucosidase, interfering with its enzymatic activity and decreasing the conversion of complex carbohydrates into glucose.

By inhibiting the enzymes α-amylase and β-glucosidase, green-synthesized AgNPs can regulate blood sugar levels by blocking them. This is achieved by lowering the rapid rate at which carbohydrates are digested and glucose is absorbed [27]. These properties make them favorable candidates for the development of environmentally friendly antidiabetic medicines. The study found that AgNPs synthesized using* Lonicera japonica* leaf extract inhibited α-amylase and β-glucosidase. A previous report showed that *Z. officinale*-mediated Ni-NPs have an inhibition effect against α-amylase and β-glucosidase, which could be a promising option for future antidiabetic therapies [28]. Mohan et al. found that the extract of *Artabotrys suaveolens *effectively suppressed the activities of α-amylase and β-glucosidase enzymes, showing comparable efficacy to that of acarbose, which was used as a control [29]. Similarly, the *A. suaveolens *extract did not exhibit any cytotoxic effects. This finding indicates that the methanolic extract of *A. suaveolens* possesses potent antidiabetic activities and could potentially be used as a nutraceutical for the treatment of diabetes mellitus.

Limitations

The green-synthesized AgNPs using* Z. officinale *and *O. gratissimum* are hindered by difficulties in standardization and clinical translation. Variability in plant extracts can impact the consistency of NPs, and the results obtained in vitro may not accurately represent the effectiveness of the NPs in living organisms. In addition, thorough safety evaluations are necessary to guarantee the effectiveness of anti-inflammatory and antidiabetic treatments, emphasizing the necessity for additional careful study to overcome these issues.

## Conclusions

The utilization of *Z. officinale *and *O. gratissimum* in synthesizing AgNPs via green methods holds promise in therapeutic nanotechnology. This biogenic approach not only ensures sustainable production but also imparts anti-inflammatory and antidiabetic properties to the NPs, attributed to bioactive compounds in plant extracts. These compounds act as both reducing and stabilizing agents during AgNP synthesis. The resulting NPs demonstrate efficacy in inhibiting key enzymes linked to inflammation and diabetes, suggesting potential therapeutic use. It is essential to conduct further research on the therapeutic applications and safety profiles of AgNPs to effectively apply these discoveries to improve health outcomes.
